# Preterm birth and sustained inflammation: consequences for the neonate

**DOI:** 10.1007/s00281-020-00803-2

**Published:** 2020-07-13

**Authors:** Alexander Humberg, Ingmar Fortmann, Bastian Siller, Matthias Volkmar Kopp, Egbert Herting, Wolfgang Göpel, Christoph Härtel, German Neonatal Network, German Center for Lung Research and Priming Immunity at the beginning of life (PRIMAL) Consortium

**Affiliations:** 1 Department of Pediatrics, University of Lübeck, Lübeck, Germany; 2 Ingmar Fortmann and Christoph Härtel (DZIF), Partner Site Hamburg-Lübeck-Borstel-Riems, Lübeck, Germany; 3 Department of Pediatrics, University of Bern, Bern, Switzerland; 4 Department of Pediatrics, University of Würzburg, Josef-Schneider-Strasse 2, 97080 Würzburg, Germany

**Keywords:** Preterm infants, Sustained inflammation, Sepsis, Microbiome, Neurocognitive outcome, Chronic pulmonary insufficiency of prematurity

## Abstract

Almost half of all preterm births are caused or triggered by an inflammatory process at the feto-maternal interface resulting in preterm labor or rupture of membranes with or without chorioamnionitis (“first inflammatory hit”). Preterm babies have highly vulnerable body surfaces and immature organ systems. They are postnatally confronted with a drastically altered antigen exposure including hospital-specific microbes, artificial devices, drugs, nutritional antigens, and hypoxia or hyperoxia (“second inflammatory hit”). This is of particular importance to extremely preterm infants born before 28 weeks, as they have not experienced important “third-trimester” adaptation processes to tolerate maternal and self-antigens. Instead of a balanced adaptation to extrauterine life, the delicate co-regulation between immune defense mechanisms and immunosuppression (tolerance) to allow microbiome establishment is therefore often disturbed. Hence, preterm infants are predisposed to sepsis but also to several injurious conditions that can contribute to the onset or perpetuation of sustained inflammation (SI). This is a continuing challenge to clinicians involved in the care of preterm infants, as SI is regarded as a crucial mediator for mortality and the development of morbidities in preterm infants. This review will outline the (i) role of inflammation for short-term consequences of preterm birth and (ii) the effect of SI on organ development and long-term outcome.

## Determinants of outcome after preterm birth

**Fig. 1:**
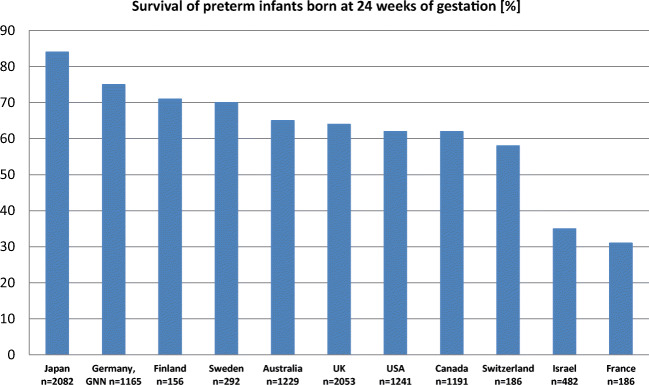
Survival rates of preterm infants born at 24 weeks of gestation in different high-income countries [1–4]; GNN German Neonatal Network

The main driver for the success of modern neonatology was the overarching aim to reduce preterm infant mortality rates. In the last decades, this goal has been achieved in many high-income countries mainly due to the significant progress in the perinatal management of high-risk pregnancies and the recent advances in neonatal intensive care. Current international network data indicate that preterm babies even at the margin of viability of 24 weeks of gestation survive in more than > 70% Fig. 1. Increasing survival of highly vulnerable babies underscores the need to assess and implement care that prevents adverse short-term complications and optimizes long-term outcomes. In Table 1, the incidences of typical adverse short-term outcomes after preterm birth are summarized. It should be noted that the current scientific evidence is primarily related to extremely preterm infants < 28 weeks of gestation (EPI; 0.3–0.6% of births in high-income countries) and to a lesser extent to very preterm infants (VPI; 28–32 weeks of gestation, 0.5–1.5% of births). The numerical majority of preterm babies—the cohort of moderate-to-late preterm infants (MLPI; 33–36 6/7 weeks of gestation; 5.5–7% of births)—has not been studied in great detail yet [5, 6].

**Table 1:** Incidences of major short-term complications of preterm birth

Outcome	EPI < 28 weeks	VPI 28–32 weeks	MPI/LPI 33–< 37 weeks	References
Intracerebral hemorrhage				
All	15–25%	1–4%	1–2%	[5–13]
Grade III–IV (Papile)	3–6%	1–2%	< 1%	
PVL	2–8%	1–6%	?	
Sepsis				
Clinical	25–60%	10–30%	5–9%	[5, 6, 8, 9, 13–15]
Blood culture confirmed	15–50%	2–6%	1–3%	[7, 8, 13, 14]
EOS	1–1.5%	0.1–0.3%	0.1–0.2%	[13, 14]
LOS	15–50%	1.5–6%	1–3%	[13–15]
NEC requiring surgery	4–10%	0.5–3%	< 1%	[6–13, 16]
SIP requiring surgery	3–8%	< 1%	< 1%	[6, 13]
Pneumothorax	4–7%	1–4%	1–2%	[5–8, 13]
BPD	15–50%	5–25%	?	[13]
ROP	2–5%	1–3%	?	[13]
Death in hospital	10–20%	2–5%	1%	[5, 6, 9–13]

*PVL* periventricular leukomalacia, *EOS* early-onset sepsis (≤ 72 h of age), *LOS* late-onset sepsis (> 72 h of age), *NEC* necrotizing enterocolitis, *SIP* spontaneous intestinal perforation, *ROP* retinopathy of prematurity, *BPD* bronchopulmonary dysplasia

**Fig. 2:**
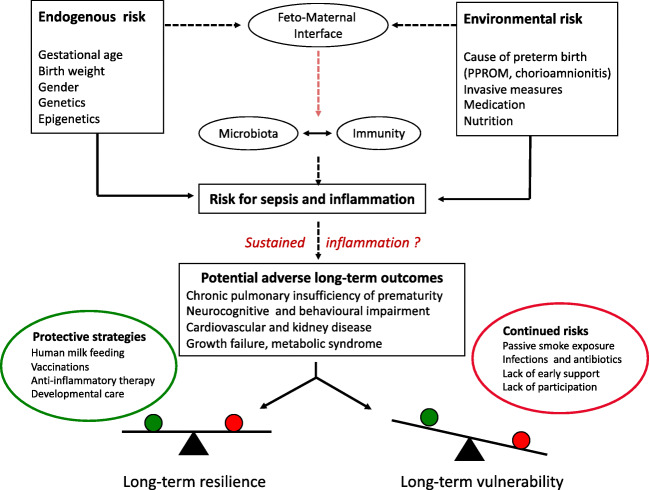
Complex risk profile of preterm infants for sustained inflammation and long-term vulnerability. This simplified model depicts that preterm infants are at risk for sustained inflammation by the virtue of their immaturity and several environmental exposures (“inflammatory” hits). The neonatal immunity is primed by the feto-maternal interface and interacts with the yet unstable microbiome. A delicate balance is needed between tolerating microbiological colonization and adequate immune responses to invasive pathogens. The neonatal “inflammatory phenotype” may result from a disturbed immune-microbiome development. The acute inflammatory process often fails to be properly resolved after clinical recovery with the consequence of sustained inflammation. The cross talk between immunity and microbiota continues and is proposed to affect developmental trajectories and long-term outcomes. Longitudinal studies are needed to account for protective modulators and continued risks for dysregulatory influences. PPROM, preterm premature rupture of membranes

The etiology of adverse outcomes is multifactorial with inflammatory processes being a crucial driving force. Inflammation is particularly important for the development of bronchopulmonary dysplasia (BPD), necrotizing enterocolitis (NEC), and retinopathy of prematurity (ROP) but also for intracerebral hemorrhage (ICH) and periventricular leukomalacia (PVL). Classical “inflammatory phenotypes” are seen in preterm infants with sepsis or NEC which are still the second most common causes of death in preterm infants after respiratory failure [4]. Acute inflammatory processes, specifically in survivors from sepsis or NEC, may not be well resolved and therefore result in SI. As outlined in Fig. 2, the main endogenous risk factors for SI are gestational age, birth weight, and gender. Genetic background is proposed to play a role for SI; however, in the specific situation of preterm infants, no single candidate variant has yet been confirmed as risk factor [17]. Thus, SI risk might represent a mixture of individual predispositions, which are the cause of preterm birth or its consequence. Due to the care under highly controlled conditions, the distinct cohort of preterm infants can serve as a model to disentangle the impact of (epi-)genetic factors from environmental influences on the development of SI. Longitudinal studies could therefore pioneer the investigation of entities in adulthood that are mediated by prolonged inflammation (e.g., chronic lung disease, coronary heart disease, neurodegenerative disease, metabolic syndrome) [18].

It is an overarching hypothesis of this narrative review that SI contributes to long-term complications of several organ systems including central nervous system (CNS) (e.g., cerebral palsy, neurobehavioral impairment), lung (chronic pulmonary insufficiency of prematurity, CPIP) [19, 20], and gut [21–24] (Fig. 2). A clear relationship has not yet been demonstrated, while indirect evidence comes from observational data. For example, the follow-up examination of GNN infants at 5 years of age (*n* = 1552) noted that neonatal sepsis (*n* = 538) is associated with a twofold risk to have a Wechsler Preschool and Primary Scale of Intelligence (WPPSI) score < 85 as compared with unaffected preterm infants (28.1 vs 12.7%, *p* < 0.001; adjusted for gestational age, intracerebral hemorrhage, and maternal educational level: OR 1.92 (95% CI: 1.40–2.64)). Hence, there is an urgent need to prevent sepsis but also to evaluate new resolution strategies of SI in order to promote long-term health in preterm infants. The major challenge, however, is the time lag between “a window of opportunity” (usually within the first 4 weeks of life when most postnatal inflammatory episodes occur) [25] and the time-point at which relevant determinants of outcome can be properly assessed (i.e., lung function, intelligence tests at school age).

## Sustained inflammation in the context of preterm birth

Preterm infants have a remarkably different system of immune regulation as compared with term infants and adults. For the immune defense, the preterm infant relies on the non-specific innate immunity, while T cell responses including those T helper cells counter-regulating inflammation, e.g., regulatory T cells (T_reg_), might be less functional. Hence, pro-inflammatory cytokine responses (e.g., interleukin (IL)-1, IL-6, IL-8, tumor necrosis factor (TNF)-a) and other inflammation-related proteins (e.g., C-reactive protein (CRP), intercellular cellular adhesion molecule (ICAM)-1, erythropoietin, ferritin) are overexpressed and insufficiently balanced by immunosuppressive elements (e.g., IL-10, S100 A8/9, myeloid-derived suppressor cells, T_reg_, CD71+ cells). In inflammation-related protein measurements in blood spots, serum and plasma are therefore used as surrogate markers to define SI in preterm babies, as outlined in several reports of the multicenter Extremely Low Gestational Age Newborn Study (ELGAN, infants < 28 weeks of gestation) group [26–29]. There is, however, a certain ambiguity in the definition of SI. Elevated concentrations of inflammation markers on two time-points within an interval of 1 week may reflect an ongoing process with failure of inflammation resolution (as we define SI in this review). Alternatively, flare-ups of a recent acute inflammatory process or two separate episodes of inflammation may be associated with elevated concentrations of inflammatory markers and cannot be discriminated from SI [30]. Hence, follow-up examinations are needed to ascertain SI in a clinical context. Notably, inflammatory processes cannot only be sustained over a long time in preterm infants but also in term infants who survived neonatal encephalopathy [31].

Recent developments in the molecular analysis of complex biomaterials suggest that there are multiple dimensions to SI in preterm infants, i.e., a disturbed interaction between the immune system and the microbiota (see also Fig. 2). The “healthy” microbiome implies a symbiotic life of the host with “friendly” microbes which provides homeostasis and protection from adverse short-term outcome. In preterm infants, however, there is evidence that (i) systemic inflammation (sepsis) often originates from the gut, (ii) the microbiota of preterm infants develops in a highly dynamic fashion and is therefore prone to dysbiosis, an imbalance with reduced microbial diversity and deficient metabolic capacity to control potential pathogens (“enemies”), and (iii) most circulating metabolic compounds (with potential to perpetuate inflammation) are actually derived from gut bacteria [32–35]. Specific diseases, such as sepsis and NEC, are preceded by gut dysbiosis and immunological dysbalance [36, 37].

Microorganisms perform essential functions mechanistically linked to the immune system of the preterm infant. Metabolites (e.g., short-chain fatty acids, SCFAs) and microbe-associated molecular patterns (MAMPs, e.g., lipopolysaccharide (LPS), peptidoglycan, flagellin) are supposed to play an important role as mediators. SCFAs such as butyrate are able to generate and enhance the pool of regulatory T cells which are capable to suppress inflammation [38]. Butyrate can also restore anti-inflammatory cytokine expression IL-10 levels by inhibiting the histone deacetylase in myeloid-derived suppressor cells (MDSCs) which limits inflammation in a *K. pneumoniae* murine sepsis model [39]. Translational studies with adult cohorts demonstrate that the composition of the gut microbiome is a crucial determinant for ex vivo cytokine production capacity and that the abundance of SCFA-producing bacteria in the gut is associated with a decreased infection risk following allogenic stem cell transplantation [40, 41]. In preterm infants, the potential link between dysbiosis-sustained inflammation and long-term outcome has not been evaluated yet [42]. A recent study in preterm babies < 32 weeks suggested that a subgroup of infants is capable of rapidly acquiring adequate immune functionality, independently of the developing heterogeneous microbiome. Preterm infants who had an inflammatory insult, however, have reduced percentages of CXCL8 but comparable levels of TNF-producing T cells, as precursors of adverse outcome. Hence, distinct identifiable differences in functionality may predict subsequent infection-mediated outcomes [43]. Intriguingly, there is evidence that the developing organ systems at risk for long-term sequelae have a significant cross talk via the microbiome-immune interaction (gut-brain axis, gut-lung axis, gut-heart axis) [44]. This emerging field of research offers new targets for microbiota stabilization in order to strengthen the ability to downregulate or resolve immune responses.

## Sustained inflammation and long-term outcome of developing organ systems

There are strong arguments for SI contributing to systemic alterations on the immune system itself but also on the tissue-/organ-specific development of organ systems. The experimental evidence for a causal relationship are often lacking, while observational studies provide indirect evidence. With regard to long-term immunological vulnerability, the risk of rehospitalization due to infections during childhood is inversely correlated with gestational age [53, 54]. Other association studies suggest a potential role of accelerated immunological aging in ex-preterm infants as indicated by telomere length differences in comparison with term-born infants [55]. On the other hand, preterm infants have a reduced risk of developing immune-mediated atopic dermatitis [56]. As potential mechanisms have currently been discussed a reduced exposure to environmental antigens, the early weaning from human milk and the premature development of a skin microbiome which can trigger specific local immune reactions to prevent dermatitis. Most of these data are case-control designs. A careful interpretation is needed, and the potential effect size of SI requires adjustment for other confounding factors for immunological susceptibility later in life including small anatomy and organ-specific predispositions which are outlined below.

### Inflammation and the preterm brain

In recent years, it has been demonstrated that the brain of preterm infants responds differently to injurious exposures than the brain of term neonates or children [57–63].

**Fig. 3:**
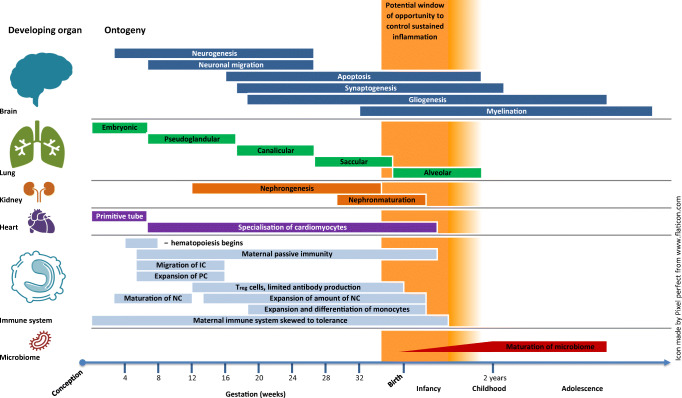
Ontogeny and potential impact of sustained inflammation on the development of the brain, lungs, kidneys, and immune system. IC, immune cells; PC, progenitor cells; NC, neutrophil cells; adapted from [45–52]

This explains the high susceptibility of extremely preterm infants to neurodevelopmental impairment and cerebral palsy [64]. Other studies indicate that also moderate and late preterm children exhibit a risk for developmental delay, most marked in the language domain, at 2 years, and behavioral problems at 7 years [65–67]. The CNS is an immune-privileged organ. However, inflammatory hits such as maternal immune activation (MIA) during pregnancy or other exposures during vulnerable periods of development (Fig. 3) can severely affect the development of the CNS [68, 69]. Three main cell types account for neuroinflammation in the human brain: microglia, astrocytes, and immune cells from the peripheral system migrating into the brain tissue after blood-brain barrier dysfunction. Pro-inflammatory cytokines are produced which further induce the activation, migration, and proliferation of cytotoxic T cells and natural killer cells. As a consequence, tissue damage occurs, particularly in the white matter [70]. The extent of damage apparently depends on the developmental stage as inflammatory stimulants (e.g., IL-1ß) lead to more pronounced neutrophil migration, chemokine production, and more disrupted blood-brain barrier in neonatal mice than in adult mice [71–73]. Microglial cells have an important physiological role for the regulation of neuronal apoptosis and neurogenesis and promote synaptic formation, pruning, and maturation [69, 74–79]. They also support axon fasciculation and myelinisation [80] and are important for the synaptogenesis and degradation of weak synapses. In terms of inflammatory function, the microglial cells display a plentitude of receptors for cytokines, chemokines, as well as for damage-associated molecular patterns (DAMPs), pathogen-associated molecular patterns (PAMPs), and factors of extracellular matrix [81]. Long cellular processes scan the environment for changes in brain tissue and are able to switch the cell into an activated stage [82–84]. Activated microglia transforms into macrophage-like cells which have the ability for phagocytosis [84], proliferation, and migration into the areas of injury. In preterm postmortem brains, the concentration of microglia was shown to be increased around cystic lesions indicating that an inflammatory process has not been properly resolved [85–87]. In the cerebrospinal fluid (CSF) of newborns with perinatal hypoxic-ischemic encephalopathy, significantly elevated levels of inflammatory markers derived from microglia were found and causally linked to neonatal white matter damage leading to spastic cerebral palsy [88–93].

Astrocytes display multiple functions on synapses, metabolite transfer, production of extracellular matrix, myelination, and blood-brain barrier formation. Proliferation of astrocytes starts around gestational age of 24 weeks with a peak around 26–28 weeks [84]. Astrocytes are usually important for anti-inflammatory homeostasis and microglia modulation [94]. However, after specific microglial stimuli, astrocytes may turn into inflammation triggering cells by releasing pro-inflammatory cytokines inducing brain damage [95–97]. A cytokine storm further supports the plasticity of astrocytes which remarkably contributes to SI in the brain [97].

Preterm infants with SI have adverse neurodevelopmental outcomes at 18–22 months including cerebral palsy, reduced Bayley Scales of Infant Development II testing scores, reduced mental and psychomotor development indices, and vision impairment [24]. The ELGAN study group [29] reported that sustained elevations of acute inflammatory proteins during the first 4 weeks of life were associated with a 2- to 5-fold increased risk for various impairments of intelligence quotient (IQ) and executive function. There was a measurable “dose effect” of early vs late elevations indicating that the consequent implementation of prevention strategies of infections such as breast milk feeding, less invasive care, hand hygiene, and antibiotic stewardship programs is needed to improve neurodevelopmental outcome [98–100].

Postmortem studies of preterm brains and animal sepsis studies suggested that γδ T cells were also linked with injury of the developing brain. Depletion of these cells protected mice against brain injury after induction of inflammatory cascades [101, 102]. Specialized B cells with innate-like functions, maternal antibodies (especially when blood-brain barrier is disrupted), and the complement system may play a further role in detrimental effects on brain tissue [103–106] which is reviewed elsewhere [45, 107]. A causal relationship between maternal infection and inflammation during pregnancy and adverse neonatal brain development has recently been demonstrated for Zika virus infection and microcephaly [108] but also exists for *Toxoplasma gondii*, herpes viruses, and several bacterial infections [109–122]. Animal models have suggested that maternal immune activation induces sustained inflammation in the offspring at a much higher likelihood when the offspring was additionally exposed to stress or drug use [123–125]. In the human context, infants that undergo early (prenatal) and subsequent inflammation have a higher risk for adverse neurocognitive and behavioral outcome [126–130]. Several clinical studies imply that a distortion of the gut-immune axis has an impact on neurocognitive outcome. Similar to the central nervous system, the enteric nervous system is able to release distinct signaling molecules which are key regulators in local (and systemic) inflammatory processes of the gut [131–135]. The complex gut-CNS interplay via neural, endocrine, metabolic, and immune factors is referred to as the “gut-brain axis,” but the pathways remain to be fully elucidated. However, several diseases of the gastrointestinal tract with inflammatory characteristics impact neurodevelopment and behavioral outcome [23, 135]. In preterm infants, NEC is a good example for the potential clinical relevance of a “gut-brain axis.” NEC is a devastating disease characterized by inflammation of the mucosa with subsequent necrosis of the intestinum. A multifactorial pathogenesis with immune response to non-physiologic intestinal microbiota (“dysbiosis”), immature intestinal anatomy, and increased expression of pro-inflammatory mediators is involved in the pathogenesis. Several studies have shown significant higher incidences of neurodevelopmental dysfunction [23, 136–140], and brain injury in magnetic resonance imaging [141], especially when infants were longer exposed to the inflammatory process [139, 140]. Given the major instability of the microbiome in the first months of life (Fig. 3), a multifactorial therapeutic approach by modulation of early dysbiosis via probiotics, controlled administration of antibiotics in accordance with antibiotic stewardship, and early termination of inflammation through early surgery may improve neurocognitive outcome [142, 143]. More research is needed to address the critical need for the early determination (at best pre-symptomatic) of adverse neurological outcome by potent biomarkers such as brain imaging and sensitive testing batteries.

### Inflammation and the preterm gut

The gastrointestinal tract (GI) underlies a vast transition through pregnancy and the period of preterm birth which makes it susceptible to inflammatory damage [144]. Major risk factors include immature mucosal barriers, dysfunctions of immune cells, reduced motility of the GI tract, decreased concentrations of secretory IgA and antimicrobial peptides, and an increased risk of dysbiosis and bacterial overgrowth as outlined above [145]. Several exogenic factors (e.g., chronic ischemia during pregnancy, chorioamnionitis, antibiotic exposure, parenteral nutrition) trigger dysbiosis and seem relevant for the risk of inflammation-mediated acute gut complications (e.g., NEC). In pregnancy mouse models, maternal inflammation leads to subsequent GI injury in the mucosal and submucosal layers of the gut [146] and alteration of GI epithelial cells in the offspring, which play a key role in innate immunity [147, 148]. In a human study of preterm infants, chorioamnionitis was shown to higher the incidence of late-onset sepsis and death among preterm infants and shifted the fecal microbiome of preterm infants [149]. The gut microbiota and its metabolites can influence immune functions and immune homeostasis both within the gut and systematically. Beyond the role of microbial-derived short-chain fatty acid (SCFA) and biotransformed bile acid (BA) potential immune ligands, inflammation can be mediated via epigenetic processes or by specific cell signaling receptors like GPRCs, TGR5, and FXR [150]. Intestinal permeability and bacterial translocation are important contributors of SI and, without repair of the intestinal barrier, might represent a continuous inflammatory stimulus leading to growth failure, short bowel syndrome, and a higher risk for autoimmunity (e.g., inflammatory bowel disease) after preterm birth [56, 151]. Growth failure is the main cause of rehospitalization of highly preterm infants during infancy [53]. Mechanistic models, e.g., short bowel syndrome zebrafish, show that the short bowel syndrome results in an increased expression of genes involved in inflammation, proliferation, and bile acid synthesis. Vice versa, genes in folate synthesis, gluconeogenesis, glycogenolysis, and fatty acid oxidation as well as activation in drug and steroid metabolism are downregulated [152]. These significant and complex alterations in pathways suggest that short bowel syndrome is provoked by SI and aggravated by long-term exposure to potential inflammatory compounds such as parenteral nutrition. Hence, anti-inflammatory strategies including human milk and pre-, pro-, and synbiotics are attractive interventions to target gut-mediated SI which are discussed in detail elsewhere (e.g., [153]).

### Inflammation and the preterm lung

The continuous antenatal process of lung tissue development makes survival of preterm infants possible when differentiation of the lung structures enables gas exchange. Lung development has been traditionally characterized in different stages [48], as outlined in Fig. 3, but goes far beyond birth. Especially at early developmental steps, interruptions can disturb lung maturation, leading to impairments in lung function and structure [47]. Therefore, extremely preterm infants are at high risk to develop chronic lung disease (CLD), which may be characterized as the BPD “phenotype,” i.e., oxygen need > 36 weeks, corrected age, or a CPIP which has clinical overlaps with the early childhood “asthma phenotype” [19]. A meta-analysis of 31 birth cohorts showed that preterm birth is associated with an increased risk of wheezing (OR 1.34 (95% CI: 1.25–1.43)) and school-age asthma (OR 1.40 (95% CI: 1.18–1.67)) [154]. A potential explanation is that preterm infants are more likely to be exposed to multiple risk factors for the development of the “asthma phenotype” (cesarean delivery, infections, and antibiotic use) as compared with term infants. All these risk factors are associated with a higher risk to SI [155].

Recent animal and human studies have also demonstrated cross talks between different mucosal sites, e.g., gut-lung axis [156]. Hence, microbiome-immunity interactions might be important for the development of BPD/CPIP which is indirectly supported by epidemiological data. In particular, multivariate logistic regression analysis from the GNN cohort (*n* = 2527 BPD-diagnosed infants, *n* = 12,826 unaffected controls) indicate that neonatal sepsis is an independent risk factor for BPD (odds ratio, OR 2.2, 95% CI: 1.9–2.4, *p* < 0.001). Sepsis also increases the risk for oxygen need at discharge (*n* = 432 affected infants/*n* = 11,634 controls; OR 1.68 (1.35–2.08), *p* = 0.001). In contrast, potential “stabilizer” of the immune-microbiome interaction such as nutrition with human milk and probiotic supplementation is associated with a reduced risk for BPD and respiratory infections [151]. Infants with BPD were shown to have a decreased airway microbiome diversity and higher concentrations of *Ureaplasma spp.* as compared with non-BPD infants. However, clinical and methodological heterogeneity make these data difficult to interpret [157, 158]. Furthermore, changes in the composition of the airway microbiome do not necessarily impact on inflammatory cytokine levels and might be heavily confounded by the clinical practice (e.g., exposure to antibiotic therapy, ventilation, nutrition, surrounding) [157, 159, 160].

The histological picture of CLD includes alterations of the lung structure with reduced septation, vascularization, number of alveoli, and simplified alveolar structures leading to a reduced capability of gas exchange and relevant lung function restrictions persisting into adulthood [47, 161, 162]. SI in the lung is triggered by mechanical ventilation, oxygen use, and infection. Tracheal aspirates of infants mechanically ventilated had higher concentrations of pro-inflammatory cytokines (IL-1β, IL-6, IL-8, TNF-α) [163, 164] (for review, see [47]). In a myriad of animal studies, a clear link between intrauterine inflammation and impaired fetal lung development is described [165–169]. Intra-amniotic injection of LPS resulted in changes of vascular markers and structural alterations of the preterm lung [167, 170]. In particular, intra-amniotic inflammation induced smooth muscle hypertrophy [167], decrease of alveoli number, and increase of alveolar volume [170]. Studies in fetal lambs with intra-amniotic administration of LPS prior to delivery showed effects on pulmonary hemodynamics [171], especially when time to delivery after injection of LPS was long enough for vascular remodeling [172], suggesting a link between maternal immune activation and pulmonary hypertension. Models of in utero infection further revealed decreased expression of vascular endothelial growth factor (VEGF) in lung tissue (important for alveologenesis), increased synthesis of extracellular matrix (collagen), and higher levels of inflammatory markers (IL-1β, IL-6, IL-17A) in lung tissue samples leading to inflammatory damage of the lungs [173–180]. Other authors demonstrated that intrauterine infection enhanced Th17 expression resulting in an immune modification of the lungs leading to uncontrolled inflammatory responses [181, 182]. This is in line with the finding that extremely preterm neonates with BPD had increased levels of Th17 and IL-17+ T_reg_ lymphocytes in cord blood samples as compared with unaffected controls [180].

Postnatal inflammation also plays an important role in the pathogenesis of CLD. Several postnatal factors are known to have an effect on the expression of pro-inflammatory cascades. One of the most important triggers in the pathogenesis of CLD is mechanical stretch injury via the use of invasive ventilation [183]. In a multitude of animal models, increased levels of pro-inflammatory cytokines [184–186] and inflammatory cells in the bronchoalveolar lavage fluid were found secondary to invasive ventilation [183, 184]. Cytokine levels are dependent on the duration of invasive ventilation [187], tidal volume strategy [188, 189], and the type of ventilatory support. Decreased inflammation was associated with non-invasive ventilation models [190, 191]. In the pathogenesis of CLD, early onset of inflammation processes seem to play an important role as an early cytokine storm was shown to impact the progress of chronic lung disease. In a longitudinal analysis of human serum cytokine profiles in preterm infants, infants with later diagnosis of BPD were shown to have elevated levels of IL-6, IL-8, and granulocyte-colony stimulating factor (G-CSF) at first weeks of life [192]. Using a murine BPD model, Rudloff et al. could demonstrate that early anti-inflammatory intervention on day one after birth via administration of IL-1 receptor antagonists was beneficial to protect from murine BPD as treatment initiated at day 6 [193]. In the clinical setting, studies on the administration of steroids to reduce the incidence of BPD argue for an effect of early administration (</= 7 days), but potential drug-related adverse outcomes need to be considered [194]. A major challenge is the assessment of early lung function parameters in preterm infants. The lung clearance index (LCI) measured by multiple breath washout (cumulative expired volume (CEV) divided by the functional residual capacity (FRC) at 1/40 of the starting gas SF6 concentration) might become a useful physiological test to detect obstructive lung disease, air trapping, or ventilation inhomogeneities. Future research needs to evaluate the significance of respiratory rate, lung MRI, or lung impedance tomography as functional biomarkers.

### Inflammation and the preterm heart

Cardiac dysfunction in adults is well described in the setting of sepsis and inflammation and is linked to the release of cytokines and tissue hypoxia [195, 196] (see for review [197]).

Little is known about the effects of intrauterine or early postnatal inflammation on the morphogenesis of the heart and its consequences for the preterm infant. Growth, formation, and development of the cardiomyocytes continue until birth [198] and may be potentially disrupted through inflammation as changes in the transcriptome of fetal cardiac tissue after inflammation were found [199]. Since the first description of David Barker [200], growing evidence suggests an association of preterm birth and high blood pressure, type 2 diabetes, and stroke in adulthood [201–203], but pathophysiological mechanisms remain to be determined. The fetal environment seems to play an important role in morphogenesis of the heart during pregnancy, as infants born after preeclampsia have a higher risk to develop high blood pressure and stroke in later years [204–206]. Exposure of the immature heart and vessels to a pro-inflammatory milieu [207, 208] may lead to epigenetic modifications [209, 210], perturbation in cardiac development-related genes, and changes of the activity of transcription factors linked to chronic inflammation and later atherosclerosis [211]. Furthermore, direct effects of bacterial toxins were shown to injure cardiomyocytes in vitro and reduce the number of cardiomyocytes [212–214] with subsequent loss of cardiac function [215]. Further hits during the neonatal period (infections, oxygen delivery) may potentially increase the risk for a chronic inflammatory response which may predispose for cardiovascular diseases in later life [216, 217]. In adults, toll-like receptors (TLR) play a role in septic myocardial dysfunction [218], demonstrated by a reduced dysfunction after administration of TLR4 inhibitors in experimental models [218–220]. In fetal sheep endotoxin models, an increase of TLR2 and 4 mRNA levels was also detectable, suggesting identical mechanisms in the fetus [214]. Other clinical studies of chorioamnionitis suggest effects of intra-amniotic inflammation on cardiac dysfunction [221, 222].

The heart of preterm infants is functionally and structurally immature and susceptible to the preterm environment [216]. Ex-preterm infants have increased left and right ventricular mass and altered systolic and diastolic function [204, 223]. Inflammatory processes are known to play an important role in the pathogenesis of pulmonary hypertension and therefore have an indirect effect on cardiac outcome. However, inflammation has direct effects on myocardial structure and function in preterm infants. Velten et al. could demonstrate an effect of systemic maternal inflammation followed by neonatal hyperoxia on left ventricular structure and systolic and diastolic dysfunction [224]. Therefore, inflammation in early life might play a significant role in remodeling of the cardiac structures with consecutive loss of function in later life. Associations between infection-related hospitalization in infancy and cardiovascular disease in adulthood were reported [225]. Ex-preterm infants often have restricted activities of daily living which might be associated with cardiorespiratory insufficiency and increased energy requirements (due to SI). Hence, growth failure is a critical issue. In the GNN follow-up investigation at 5 years of age, we noted that a significant proportion of preterm infants has a growth disadvantage as compared with term infants examined in the “German Health Interview and Examination Survey for Children and Adolescents” (KiGGS) study (mean ~ 2.5 kg less body weight, ~ 4 cm less body length). The administration of *Lactobacillus acidophilus/Bifidobacterium* spp. probiotics—as proposed “stabilizer of the microbiome”—during primary stay in hospital resulted in improved growth, particularly in infants who were early exposed to antibiotics [151].

### Inflammation and the preterm kidney

Little information exists on the effects of inflammation on preterm kidney function. Term newborns present usually with an amount of over 300,000 nephrons [226]. Theoretically, nephrogenesis is sensitive for inflammatory events, as it continues until 34–36 weeks of gestation [46] (Fig. 3). Susceptibility of the nephrogenesis to exogenous noxious substances was proven in experimental models with postnatal exposure to hyperoxia and a 25% reduction of nephron numbers that persisted into adulthood [227]. In adults with septicemia, oliguria is interpreted as manifestation of renal inflammation [228, 229]. Oliguria is often a hallmark of the “inflammatory phenotype” in preterm infants. Clinical data of infants born after chorioamnionitis and treated with indomethacin suggest a harming effect on renal development [230]. In preterm fetal sheep exposed to LPS-induced intrauterine inflammation, a reduction of nephron numbers, but not of kidney or birth weight, was demonstrated [231]. This might be a risk factor for impaired renal function or a predisposition for second hits (indomethacin) during the neonatal period and for hypertension in later life [232]. How these observations occur if these are the result of renal hypoperfusion or renal inflammation during chorioamnionitis and which molecular pathways lead to such damages is largely unknown and remains speculative.

### Inflammation and preterm sex differences

The personalized approach to maximize results and minimize risks particularly of new targeted treatments against SI requires genotypic and phenotypic fine-tuning of the single preterm baby including information beyond gestational age as the main contributor to adverse SI-related outcome. The significance of gender (including the role of sex hormones, sex chromosomes) has been well established for inflammation processes and microbiome development in animal models and observed in a variety of human studies [233]. The gender difference is particularly marked among preterm infants, where females have a distinct survival and outcome advantage at similar birth weights and gestational ages [4, 234]. Female preterm infants have a decreased risk for severe courses of infectious diseases as compared with males (bacterial, viral, fungal) [234–241]. Gender differences have also been reported in several responses of inflammatory signaling processes, with a tendency towards a more anti-inflammatory environment in female infants. For example, reduced oxidative stress biomarkers and concentrations of myeloperoxidase were found in female preterms [242, 243]. In umbilical cord blood, females were shown to have increased CD4+/CD8+ T cell ratios and reduced numbers of NK cells [233]. After stimulation with LPS, female cord blood cells showed reduced concentrations of pro-inflammatory cytokines (IL-1ß and IL-6) than male cells [244]. Animal models reveal a gender-specific response to inflammatory stimuli with an advantage for female rodents. In female newborn rats, for example, the increased production of IL-2, IFN-γ, and TNF-α is thought to promote the Th-1 response in better defending against infection [245]. Reasons for the mmunological advantage of female infants are mainly unknown, while multidirectional cross talk between host immunity, microbiota, and the endocrine system is anticipated. Bacteria are able to produce hormones (e.g., serotonin, dopamine, and somatostatine), to interact with host hormones (e.g., estrogens), and to regulate the host hormones’ homeostasis (e.g., by inhibiting gene prolactin transcription or converting glucocorticoids to androgens) which can lead to context-dependent immunosuppression or activation. Differences in the gut microbiome profiles in newborn infants have been described with higher abundance of *Enterobacteriales* and lower abundance of *Clostridiales* in males shortly after birth [246].

Some light is shed on the important role of sex chromosomes, signaling genes and single nucleotide polymorphisms, microRNAs, methylation alterations, and hormonal concentrations which are discussed elsewhere [247]. The role of these findings on clinical effects is unknown and requires further studies.

## Conclusions and future outlook

The concept that sustained inflammation contributes to short- and long-term complications after preterm birth has been largely accepted. Numerous observational studies demonstrate a risk association between unfavorable immune adaptation and long-term vulnerability for infections [248], CPIP and asthma [56, 249], neurodevelopmental impairment, and stress incontinence [250, 251]. The underlying mechanisms, in particular the host factors determining resilience or long-term vulnerability, remain to be elucidated. To improve outcome in preterm babies, clinicians hope for the successful identification of distinct “immune-microbiome signatures” in preterm babies as biomarkers for the development of new SI-targeted therapies. The main challenges and potential approaches are described in Table 2. At this stage, non-specific preventive measures of acute inflammatory episodes and SI have the highest priority in clinical practice, including the following:

(i) Hygiene and surveillance: the key to reduce sepsis rates is hand hygiene, training of staff and parents, surveillance and network participation, and the avoidance of understaffing and overcrowding.
(ii) Less invasive care approach: preterm infants may benefit from less invasive respiratory management [13, 252] and rapid feeding advancement [253].
(iii) Human milk feeding: human milk is beneficial in terms of sepsis rates and reduces the risk for ROP and BPD and asthma. It contains S100 A8/9, growth factors, and human milk oligosaccharides (HMOs) which cannot be digested by humans that facilitate the growth of “beneficial” bifidobacteria and support the production of SFCAs.
(iv) Antibiotic stewardship programs: the implementation of antibiotic stewardship programs increases awareness and reduces the rates of drug-resistant organisms.
(v) Strengthening the immune system: vaccinations to mothers and infants are important modes of infection prevention and immunological maturation, and the continuum of parental care from birth even in an intensive care setting is highly immune promoting.
(vi) Avoidance of adverse exposures: extensive counseling of parents is needed to avoid passive smoking and stressful experiences.
(vii) Provision of developmental care: early neurodevelopmental support and continued case management are needed to promote long-term health.

**Table 2:** Targeting sustained inflammation to improve outcome in preterm infants: challenges and outcomes

Challenge	Approach
Inflammatory episodes (sepsis, NEC) remain predominant causes of mortality and long-term morbidity Large center-specific variations	Implementation of inflammation prevention bundles (hygiene, antibiotic stewardship programs, restrictive use of invasive measures, promotion of human milk feeding) Continuous establishment of quality improvement networks
Understanding of underlying pathophysiological mechanisms Availability of animal models for preterm infants Limited opportunity to study tissue-specific aspects	Well-phenotyped large-scale longitudinal studies, systems biology approaches Mechanistic neonatal mouse or rhesus monkey models, in silico modeling Organoid models to investigate organ-specific mechanisms of SI
Disentangle the impact of prenatal and postnatal factors	Linking perinatal, neonatal datasets to follow-up data from cohort studies; target “neonatal window of opportunity”
Establishment of a physiological immune-microbiome adaptation despite postnatal intensive care	Basic research addressing long-term effects of perinatal exposures (e.g., antibiotics), postnatal biomarkers (e.g., S100 A8/9, T_reg_), and interventions (pre-/pro-/synbiotics; anti-inflammatory compounds; stem cells) Phase I–III clinical trials and randomized, placebo controlled trials with long-term follow-up
Lack of valid outcome measures of important organ functions (e.g., cognition, lung function)	Development of new tools for early short-term assessment; childhood follow-up with detailed determination of beneficial factors (human milk feeding, vaccinations) and harmful exposures (passive smoke, lack of participation) Define further “windows of opportunity” during infancy and childhood; study interventions to promote long-term health in controlled trials (e.g., music, sport, nutrition)
Targeted personalized therapies of preterm infants	Use of polygenic risk scores from adult cohorts for preterm infants and establishment of valuable trajectory-specific biomarkers (e.g., S100 A8/9)

Hence, a more personalized precision medicine approach is needed. The neonatal period has lifelong imprinting effects and remains the primary window of opportunity for prevention or intervention [254]. It could be promising to extrapolate the set trajectory of immune development in term infants [255] into the context of preterm birth. A proportion of preterm babies is capable of rapid neonatal progression to “catch up” immune function with immune profiles converging in a similar time frame to term babies [256]. Infants with postnatal inflammation, however, may have distinct immune signatures and microbiome patterns before the “inflammatory” event and thereafter. Polygenic risk profiling can identify several stages of vulnerability, and standardized observational times can target gene-environment interactions. Systems biology approaches and large sample sizes are needed to account for the different factors which challenge the transcriptional and epigenetic reprogramming of systemic and mucosal immunity, e.g., colonizing microbes and their metabolites, nutritional antigens, drugs, and vaccines [257, 258]. Multinational network approaches are needed to align definitions for sepsis and sustained inflammation and to study interventions in pre-specified subgroups.

## Abbreviations

BA: Bile acid
BPD: Bronchopulmonary dysplasia
CI: Confidence interval
CNS: Central nervous system
CPIP: Chronic pulmonary insufficiency of prematurity
CRP: C-reactive protein
CSF: Cerebrospinal fluid
DAMP: Damage-associated molecular patterns
ELGAN: Extremely Low Gestational Age Newborn Study
EOS: Early-onset sepsis
EPI: Extremely preterm infants
FXR: Farnesol X receptor
G-CSF: Granulocyte-colony stimulating factor
GI: Gastrointestinal tract
GNN: German Neonatal Network
GPRC: G protein-coupled receptor
ICAM: Intercellular cellular adhesion molecule
ICH: Intracerebral hemorrhage
IL: Interleukin
IQ: Intelligence quotient
KiGGS: German Health Interview and Examination Survey for Children and Adolescents
LOS: Late-onset sepsis
LPI: Late preterm infants
LPS: Lipopolysaccharide
MAMP: Microbe-associated molecular patterns
MDSCs: Myeloid-derived suppressor cells
MIA: Maternal immune activation
MLPI: Moderate and late preterm infants
MPI: Moderate preterm infants
NEC: Necrotizing enterocolitis
OR: Odds ratio
PAMP: Pathogen-associated molecular patterns
PPROM: Preterm premature rupture of membranes
PVL: Periventricular leukomalacia
ROP: Retinopathy of prematurity
SCFA: Short-chain fatty acids
SI: Sustained inflammation
TLR: Toll-like receptors
TNF: Tumor necrosis factor
T_reg_: Regulatory T cells
VEGF: Vascular endothelial growth factor
VPI: Very preterm infants
WPPSI: Wechsler Preschool and Primary Scale of Intelligence

## References

[1] Helenius K, Sjörs G, Shah PS (2017). Survival in very preterm infants: an international comparison of 10 National Neonatal Networks. Pediatrics.

[2] Stoll BJ, Hansen NI, Bell EF (2015). Trends in care practices, morbidity, and mortality of extremely preterm neonates, 1993-2012. JAMA.

[3] Ancel P-Y, Goffinet F, Kuhn P et al (2015) Survival and morbidity of preterm children born at 22 through 34 weeks’ gestation in France in 2011. JAMA Pediatr. 10.1001/jamapediatrics.2014.3351 10.1001/jamapediatrics.2014.3351 25621457

[4] Humberg A, Härtel C, Rausch TK (2020). Active perinatal care of preterm infants in the German Neonatal Network. Arch Dis Child Fetal Neonatal Ed.

[5] Poets C, Wallwiener D, Vetter K (2012). Risks associated with delivering infants 2 to 6 weeks before term-a review of recent data. Dtsch Arztebl Int.

[6] Natarajan G, Shankaran S (2016). Short- and long-term outcomes of moderate and late preterm infants. Am J Perinatol.

[7] Kirkby S, Greenspan JS, Kornhauser M, Schneiderman R (2007). Clinical outcomes and cost of the moderately preterm infant. Adv Neonatal Care.

[8] Escobar GJ, McCormick MC, Zupancic JAF (2006). Unstudied infants: outcomes of moderately premature infants in the neonatal intensive care unit. Arch Dis Child Fetal Neonatal Ed.

[9] Altman M, Vanpée M, Cnattingius S, Norman M (2011) Neonatal morbidity in moderately preterm infants: a Swedish national population-based study. J Pediatr 158:239–44.e1. 10.1016/j.jpeds.2010.07.047 10.1016/j.jpeds.2010.07.047 20828716

[10] Trembath A, Payne A, Colaizy T (2016). The problems of moderate preterm infants. Semin Perinatol.

[11] Walsh MC, Bell EF, Kandefer S (2017). Neonatal outcomes of moderately preterm infants compared to extremely preterm infants. Pediatr Res.

[12] Bajaj M, Natarajan G, Shankaran S, et al (2018) Delivery room resuscitation and short-term outcomes in moderately preterm infants. J Pediatr 195:33-38.e2. 10.1016/j.jpeds.2017.11.039 10.1016/j.jpeds.2017.11.039 PMC5869086 29306493

[13] Härtel C, Paul P, Hanke K et al (2018) Less invasive surfactant administration and complications of preterm birth. Sci Rep 8. 10.1038/s41598-018-26437-x 10.1038/s41598-018-26437-x PMC5974027 29844331

[14] Stoll BJ, Hansen NI, Sánchez PJ (2011). Early onset neonatal sepsis: the burden of group B streptococcal and E. coli disease continues. Pediatrics.

[15] Boyle EM, Johnson S, Manktelow B (2015). Neonatal outcomes and delivery of care for infants born late preterm or moderately preterm: a prospective population-based study. Arch Dis Child Fetal Neonatal Ed.

[16] Battersby C, Santhalingam T, Costeloe K, Modi N (2018). Incidence of neonatal necrotising enterocolitis in high-income countries: a systematic review. Arch Dis Child Fetal Neonatal Ed.

[17] Göpel W, Westermann E, Pagel F (2018). The genetic background of neonatal disease. Neonatology.

[18] Tabas I, Glass CK (2013). Anti-inflammatory therapy in chronic disease: challenges and opportunities. Science.

[19] Keller RL, Feng R, DeMauro SB, et al (2017) Bronchopulmonary dysplasia and perinatal characteristics predict 1-year respiratory outcomes in newborns born at extremely low gestational age: a prospective cohort study. J Pediatr 187:89-97.e3. 10.1016/j.jpeds.2017.04.026 10.1016/j.jpeds.2017.04.026 PMC5533632 28528221

[20] Steinhorn R, Davis JM, Göpel W, et al (2017) Chronic pulmonary insufficiency of prematurity: developing optimal endpoints for drug development. J Pediatr 191:15-21.e1. 10.1016/j.jpeds.2017.08.006 10.1016/j.jpeds.2017.08.006 29173299

[21] Agrawal S, Rao SC, Bulsara MK, Patole SK (2018). Prevalence of autism spectrum disorder in preterm infants: a meta-analysis. Pediatrics.

[22] Ardalan M, Chumak T, Vexler Z, Mallard C (2019). Sex-dependent effects of perinatal inflammation on the brain: implication for neuro-psychiatric disorders. Int J Mol Sci.

[23] Humberg A, Spiegler J, Fortmann MI et al (2020) Surgical necrotizing enterocolitis but not spontaneous intestinal perforation is associated with adverse neurological outcome at school age Sci Rep:10. 10.1038/s41598-020-58761-6 10.1038/s41598-020-58761-6 PMC7012917 32047169

[24] Stoll BJ, Hansen NI, Adams-Chapman I (2004). Neurodevelopmental and growth impairment among extremely low-birth-weight infants with neonatal infection. JAMA.

[25] Boghossian NS, Page GP, Bell EF, et al (2013) Late-onset sepsis in very low birth weight infants from singleton and multiple-gestation births. J Pediatr 162:1120–4, 1124.e1. 10.1016/j.jpeds.2012.11.089 10.1016/j.jpeds.2012.11.089 PMC3633723 23324523

[26] Leviton A, Kuban K, O’Shea TM, et al (2011) The relationship between early concentrations of 25 blood proteins and cerebral white matter injury in preterm newborns: the ELGAN study. J Pediatr 158:897-903.e1–5. 10.1016/j.jpeds.2010.11.059 10.1016/j.jpeds.2010.11.059 21238986

[27] O’Shea TM, Allred EN, Kuban KCK, et al (2012) Elevated concentrations of inflammation-related proteins in postnatal blood predict severe developmental delay at 2 years of age in extremely preterm infants. J Pediatr 160:395-401.e4. 10.1016/j.jpeds.2011.08.069 10.1016/j.jpeds.2011.08.069 PMC3279610 22000304

[28] Dammann O, Leviton A (2014). Intermittent or sustained systemic inflammation and the preterm brain. Pediatr Res.

[29] Kuban KCK, Joseph RM, O’Shea TM, et al (2017) Circulating inflammatory-associated proteins in the first month of life and cognitive impairment at age 10 years in children born extremely preterm. J Pediatr 180:116-123.e1. 10.1016/j.jpeds.2016.09.054 10.1016/j.jpeds.2016.09.054 PMC5183478 27788929

[30] Dammann O, Allred EN, Fichorova RN (2016). Duration of systemic inflammation in the first postnatal month among infants born before the 28th week of gestation. Inflammation.

[31] Zareen Z, Strickland T, Eneaney VM (2020). Cytokine dysregulation persists in childhood post neonatal encephalopathy. BMC Neurol.

[32] Pammi M, Cope J, Tarr PI (2017). Intestinal dysbiosis in preterm infants preceding necrotizing enterocolitis: a systematic review and meta-analysis. Microbiome.

[33] Rusconi B, Jiang X, Sidhu R et al (2018) Gut sphingolipid composition as a prelude to necrotizing enterocolitis. Sci Rep 8. 10.1038/s41598-018-28862-4 10.1038/s41598-018-28862-4 PMC6054655 30030452

[34] Baumann-Dudenhoeffer AM, D’Souza AW, Tarr PI (2018). Infant diet and maternal gestational weight gain predict early metabolic maturation of gut microbiomes. Nat Med.

[35] Graspeuntner S, Waschina S, Künzel S (2019). Gut dysbiosis with bacilli dominance and accumulation of fermentation products precedes late-onset sepsis in preterm infants. Clin Infect Dis.

[36] Tarr PI, Warner BB (2016). Gut bacteria and late-onset neonatal bloodstream infections in preterm infants. Semin Fetal Neonatal Med.

[37] Warner BB, Deych E, Zhou Y (2016). Gut bacteria dysbiosis and necrotising enterocolitis in very low birthweight infants: a prospective case-control study. Lancet.

[38] Arpaia N, Campbell C, Fan X (2013). Metabolites produced by commensal bacteria promote peripheral regulatory T-cell generation. Nature.

[39] Chakraborty K, Raundhal M, Chen BB et al (2017) The mito-DAMP cardiolipin blocks IL-10 production causing persistent inflammation during bacterial pneumonia. Nat Commun 8. 10.1038/ncomms13944 10.1038/ncomms13944 PMC5241690 28074841

[40] Schirmer M, Smeekens SP, Vlamakis H, et al (2016) Linking the human gut microbiome to inflammatory cytokine production capacity. Cell 167:1125-1136.e8. 10.1016/j.cell.2016.10.020 10.1016/j.cell.2016.10.020 PMC5131922 27814509

[41] Haak BW, Littmann ER, Chaubard J-L (2018). Impact of gut colonization with butyrate-producing microbiota on respiratory viral infection following allo-HCT. Blood.

[42] Groer MW, Gregory KE, Louis-Jacques A (2015). The very low birth weight infant microbiome and childhood health. Birth Defects Res C Embryo Today.

[43] Kamdar S, Hutchinson R, Laing A et al (2020) Perinatal inflammation influences but does not arrest rapid immune development in preterm babies. Nat Commun 11. 10.1038/s41467-020-14923-8 10.1038/s41467-020-14923-8 PMC7062833 32152273

[44] Tirone C, Pezza L, Paladini A et al (2019) Gut and lung microbiota in preterm infants: immunological modulation and implication in neonatal outcomes. Front Immunol 10. 10.3389/fimmu.2019.02910 10.3389/fimmu.2019.02910 PMC6920179 31921169

[45] Estes ML, McAllister AK (2016) Maternal immune activation: implications for neuropsychiatric disorders. Science (80- ) 353:772–777. 10.1126/science.aag3194 10.1126/science.aag3194 PMC5650490 27540164

[46] Mackenzie HS, Brenner BM (1995). Fewer nephrons at birth: a missing link in the etiology of essential hypertension?. Am J Kidney Dis.

[47] Shahzad T, Radajewski S, Chao C-M (2016). Pathogenesis of bronchopulmonary dysplasia: when inflammation meets organ development. Mol Cell Pediatr.

[48] Joshi S, Kotecha S (2007). Lung growth and development. Early Hum Dev.

[49] Dowling DJ, Levy O (2014). Ontogeny of early life immunity. Trends Immunol.

[50] Puddu M, Fanos V, Podda F, Zaffanello M (2009). The kidney from prenatal to adult life: perinatal programming and reduction of number of nephrons during development. Am J Nephrol.

[51] Clancy B, Kersh B, Hyde J (2007). Web-based method for translating neurodevelopment from laboratory species to humans. Neuroinformatics.

[52] Simon AK, Hollander GA, McMichael A (2015) Evolution of the immune system in humans from infancy to old age. Proc R Soc B Biol Sci 282 10.1098/rspb.2014.3085 PMC4707740 26702035

[53] Srinivasjois R, Slimings C, Einarsdóttir K (2015). Association of gestational age at birth with reasons for subsequent hospitalisation: 18 years of follow-up in a Western Australian population study. PLoS One.

[54] Miller JE, Hammond GC, Strunk T (2016). Association of gestational age and growth measures at birth with infection-related admissions to hospital throughout childhood: a population-based, data-linkage study from Western Australia. Lancet Infect Dis.

[55] Smeets CCJ, Codd V, Samani NJ, Hokken-Koelega ACS (2015). Leukocyte telomere length in young adults born preterm: support for accelerated biological ageing. PLoS One.

[56] Goedicke-Fritz S, Härtel C, Krasteva-Christ G (2017). Preterm birth affects the risk of developing immune-mediated diseases. Front Immunol.

[57] Babikian T, Prins ML, Cai Y (2010). Molecular and physiological responses to juvenile traumatic brain injury: focus on growth and metabolism. Dev Neurosci.

[58] Giza CC, Mink RB, Madikians A (2007). Pediatric traumatic brain injury: not just little adults. Curr Opin Crit Care.

[59] Claus CP, Tsuru-Aoyagi K, Adwanikar H (2010). Age is a determinant of leukocyte infiltration and loss of cortical volume after traumatic brain injury. Dev Neurosci.

[60] Potts MB, Koh S-E, Whetstone WD (2006). Traumatic injury to the immature brain: inflammation, oxidative injury, and iron-mediated damage as potential therapeutic targets. NeuroRx.

[61] Qiu L, Zhu C, Wang X (2007). Less neurogenesis and inflammation in the immature than in the juvenile brain after cerebral hypoxia-ischemia. J Cereb Blood Flow Metab.

[62] Zhu C, Wang X, Xu F (2005). The influence of age on apoptotic and other mechanisms of cell death after cerebral hypoxia-ischemia. Cell Death Differ.

[63] Blomgren K, Leist M, Groc L (2007). Pathological apoptosis in the developing brain. Apoptosis.

[64] Adams-Chapman I, Heyne RJ, DeMauro SB et al (2018) Neurodevelopmental impairment among extremely preterm infants in the neonatal research network. Pediatrics 141. 10.1542/peds.2017-3091 10.1542/peds.2017-3091 PMC5914487 29666163

[65] Cheong JL, Doyle LW, Burnett AC (2017). Association between moderate and late preterm birth and neurodevelopment and social-emotional development at age 2 years. JAMA Pediatr.

[66] Dotinga BM, de Winter AF, Bocca-Tjeertes IFA (2019). Longitudinal growth and emotional and behavioral problems at age 7 in moderate and late preterms. PLoS One.

[67] Synnes A, Hicks M (2018). Neurodevelopmental outcomes of preterm children at school age and beyond. Clin Perinatol.

[68] Eppig C, Fincher CL, Thornhill R (2010). Parasite prevalence and the worldwide distribution of cognitive ability. Proc Biol Sci.

[69] Schafer DP, Lehrman EK, Kautzman AG (2012). Microglia sculpt postnatal neural circuits in an activity and complement-dependent manner. Neuron.

[70] Khwaja O, Volpe JJ (2008). Pathogenesis of cerebral white matter injury of prematurity. Arch Dis Child Fetal Neonatal Ed.

[71] Anthony DC, Bolton SJ, Fearn S, Perry VH (1997). Age-related effects of interleukin-1 beta on polymorphonuclear neutrophil-dependent increases in blood-brain barrier permeability in rats. Brain.

[72] Campbell SJ, Carare-Nnadi RO, Losey PH, Anthony DC (2007). Loss of the atypical inflammatory response in juvenile and aged rats. Neuropathol Appl Neurobiol.

[73] Lawson LJ, Perry VH (1995). The unique characteristics of inflammatory responses in mouse brain are acquired during postnatal development. Eur J Neurosci.

[74] Wakselman S, Béchade C, Roumier A (2008). Developmental neuronal death in hippocampus requires the microglial CD11b integrin and DAP12 immunoreceptor. J Neurosci.

[75] Cunningham CL, Martinez-Cerdeno V, Noctor SC (2013). Microglia regulate the number of neural precursor cells in the developing cerebral cortex. J Neurosci.

[76] Mazaheri F, Breus O, Durdu S (2014). Distinct roles for BAI1 and TIM-4 in the engulfment of dying neurons by microglia. Nat Commun.

[77] Shigemoto-Mogami Y, Hoshikawa K, Goldman JE (2014). Microglia enhance neurogenesis and oligodendrogenesis in the early postnatal subventricular zone. J Neurosci.

[78] Paolicelli RC, Bolasco G, Pagani F, et al (2011) Synaptic pruning by microglia is necessary for normal brain development. Science (80- ) 333:1456–1458. 10.1126/science.1202529 10.1126/science.1202529 21778362

[79] Kim H-J, Cho M-H, Shim WH (2017). Deficient autophagy in microglia impairs synaptic pruning and causes social behavioral defects. Mol Psychiatry.

[80] Wlodarczyk A, Holtman IR, Krueger M (2017). A novel microglial subset plays a key role in myelinogenesis in developing brain. EMBO J.

[81] Chiaretti A, Antonelli A, Mastrangelo A (2008). Interleukin-6 and nerve growth factor upregulation correlates with improved outcome in children with severe traumatic brain injury. J Neurotrauma.

[82] Saliba E, Henrot A (2001). Inflammatory mediators and neonatal brain damage. Neonatology.

[83] Twohig JP, Cuff SM, Yong AA, Wang ECY (2011). The role of tumor necrosis factor receptor superfamily members in mammalian brain development, function and homeostasis. Rev Neurosci.

[84] Bokobza C, Van Steenwinckel J, Mani S (2019). Neuroinflammation in preterm babies and autism spectrum disorders. Pediatr Res.

[85] Hagberg H, Peebles D, Mallard C (2002). Models of white matter injury: comparison of infectious, hypoxic-ischemic, and excitotoxic insults. Ment Retard Dev Disabil Res Rev.

[86] Hagberg H, David Edwards A, Groenendaal F (2016). Perinatal brain damage: the term infant. Neurobiol Dis.

[87] Verney C, Rees S, Biran V (2010). Neuronal damage in the preterm baboon: impact of the mode of ventilatory support. J Neuropathol Exp Neurol.

[88] Sävman K, Heyes MP, Svedin P, Karlsson A (2013). Microglia/macrophage-derived inflammatory mediators galectin-3 and quinolinic acid are elevated in cerebrospinal fluid from newborn infants after birth asphyxia. Transl Stroke Res.

[89] Dammann O, Leviton A (1997). Maternal intrauterine infection, cytokines, and brain damage in the preterm newborn. Pediatr Res.

[90] Grether JK, Nelson KB (1997). Maternal infection and cerebral palsy in infants of normal birth weight. JAMA.

[91] Hagberg H, Mallard C, Jacobsson B (2005). Role of cytokines in preterm labour and brain injury. BJOG An Int J Obstet Gynaecol.

[92] Ahlin K, Himmelmann K, Hagberg G (2013). Cerebral palsy and perinatal infection in children born at term. Obstet Gynecol.

[93] Hermansen MC, Hermansen MG (2006). Perinatal infections and cerebral palsy. Clin Perinatol.

[94] Norden DM, Fenn AM, Dugan A, Godbout JP (2014). TGFβ produced by IL-10 redirected astrocytes attenuates microglial activation. Glia.

[95] Joe E-H, Choi D-J, An J (2018). Astrocytes, microglia, and Parkinson’s disease. Exp Neurobiol.

[96] Ponath G, Park C, Pitt D (2018). The role of astrocytes in multiple sclerosis. Front Immunol.

[97] Shiow LR, Favrais G, Schirmer L (2017). Reactive astrocyte COX2-PGE2 production inhibits oligodendrocyte maturation in neonatal white matter injury. Glia.

[98] Shane AL, Stoll BJ (2014). Neonatal sepsis: progress towards improved outcomes. J Inf Secur.

[99] Davis JW, Odd D, Jary S, Luyt K (2016). The impact of a sepsis quality improvement project on neurodisability rates in very low birthweight infants. Arch Dis Child Fetal Neonatal Ed.

[100] Dong Y, Speer CP (2015). Late-onset neonatal sepsis: recent developments. Arch Dis Child Fetal Neonatal Ed.

[101] Albertsson A-M, Zhang X, Vontell R (2018). γδ T cells contribute to injury in the developing brain. Am J Pathol.

[102] Zhang X, Rocha-Ferreira E, Li T, et al (2017) γδT cells but not αβT cells contribute to sepsis-induced white matter injury and motor abnormalities in mice. J Neuroinflammation 14:255. 10.1186/s12974-017-1029-9 10.1186/s12974-017-1029-9 PMC5738716 29262837

[103] Baumgarth N (2011). The double life of a B-1 cell: self-reactivity selects for protective effector functions. Nat Rev Immunol.

[104] Upender MB, Dunn JA, Wilson SM, Naegele JR (1997). Immunoglobulin molecules are present in early-generated neuronal populations in the rat cerebral cortex and retina. J Comp Neurol.

[105] Nakahara J, Tan-Takeuchi K, Seiwa C (2003). Signaling via immunoglobulin Fc receptors induces oligodendrocyte precursor cell differentiation. Dev Cell.

[106] Nakahara J, Seiwa C, Shibuya A (2003). Expression of Fc receptor for immunoglobulin M in oligodendrocytes and myelin of mouse central nervous system. Neurosci Lett.

[107] Morimoto K, Nakajima K (2019) Role of the immune system in the development of the central nervous system. Front Neurosci 13 10.3389/fnins.2019.00916 PMC6735264 31551681

[108] Mlakar J, Korva M, Tul N (2016). Zika virus associated with microcephaly. N Engl J Med.

[109] Brown AS, Begg MD, Gravenstein S (2004). Serologic evidence of prenatal influenza in the etiology of schizophrenia. Arch Gen Psychiatry.

[110] Brown AS, Hooton J, Schaefer CA (2004). Elevated maternal interleukin-8 levels and risk of schizophrenia in adult offspring. Am J Psychiatry.

[111] Clarke MC, Tanskanen A, Huttunen M (2009). Evidence for an interaction between familial liability and prenatal exposure to infection in the causation of schizophrenia. Am J Psychiatry.

[112] Nielsen PR, Laursen TM, Mortensen PB (2013). Association between parental hospital-treated infection and the risk of schizophrenia in adolescence and early adulthood. Schizophr Bull.

[113] Atladóttir HÓ, Thorsen P, Østergaard L (2010). Maternal infection requiring hospitalization during pregnancy and autism spectrum disorders. J Autism Dev Disord.

[114] Yamashita Y, Fujimoto C, Nakajima E (2003). Possible association between congenital cytomegalovirus infection and autistic disorder. J Autism Dev Disord.

[115] Brown AS, Vinogradov S, Kremen WS (2009). Prenatal exposure to maternal infection and executive dysfunction in adult schizophrenia. Am J Psychiatry.

[116] Mednick SA, Machon RA, Huttunen MO, Bonett D (1988). Adult schizophrenia following prenatal exposure to an influenza epidemic. Arch Gen Psychiatry.

[117] Mortensen PB, Nørgaard-Pedersen B, Waltoft BL (2007). Toxoplasma gondii as a risk factor for early-onset schizophrenia: analysis of filter paper blood samples obtained at birth. Biol Psychiatry.

[118] Buka SL, Cannon TD, Torrey EF (2008). Maternal exposure to herpes simplex virus and risk of psychosis among adult offspring. Biol Psychiatry.

[119] Xiao J, Buka SL, Cannon TD (2009). Serological pattern consistent with infection with type I toxoplasma gondii in mothers and risk of psychosis among adult offspring. Microbes Infect.

[120] Mortensen PB, Pedersen CB, Hougaard DM (2010). A Danish National Birth Cohort study of maternal HSV-2 antibodies as a risk factor for schizophrenia in their offspring. Schizophr Res.

[121] Torrey EF, Bartko JJ, Lun Z-R, Yolken RH (2007). Antibodies to toxoplasma gondii in patients with schizophrenia: a meta-analysis. Schizophr Bull.

[122] Torrey EF, Bartko JJ, Yolken RH (2012). Toxoplasma gondii and other risk factors for schizophrenia: an update. Schizophr Bull.

[123] Knuesel I, Chicha L, Britschgi M (2014). Maternal immune activation and abnormal brain development across CNS disorders. Nat Rev Neurol.

[124] Reisinger S, Khan D, Kong E (2015). The poly(I:C)-induced maternal immune activation model in preclinical neuropsychiatric drug discovery. Pharmacol Ther.

[125] Meyer U (2014). Prenatal poly(i:C) exposure and other developmental immune activation models in rodent systems. Biol Psychiatry.

[126] Korzeniewski SJ, Romero R, Cortez J (2014). A “multi-hit” model of neonatal white matter injury: cumulative contributions of chronic placental inflammation, acute fetal inflammation and postnatal inflammatory events.

[127] Wang X, Hagberg H, Nie C (2007). Dual role of intrauterine immune challenge on neonatal and adult brain vulnerability to hypoxia-ischemia. J Neuropathol Exp Neurol.

[128] Eklind S, Mallard C, Leverin AL (2001). Bacterial endotoxin sensitizes the immature brain to hypoxic-ischaemic injury. Eur J Neurosci.

[129] Yang L, Sameshima H, Ikeda T, Ikenoue T (2004). Lipopolysaccharide administration enhances hypoxic-ischemic brain damage in newborn rats. J Obstet Gynaecol Res.

[130] Rousset CI, Kassem J, Olivier P (2008). Antenatal bacterial endotoxin sensitizes the immature rat brain to postnatal excitotoxic injury. J Neuropathol Exp Neurol.

[131] Lomax AE, Fernández E, Sharkey KA (2005). Plasticity of the enteric nervous system during intestinal inflammation. Neurogastroenterol Motil.

[132] Rühl A (2005). Glial cells in the gut. Neurogastroenterol Motil.

[133] Capoccia E, Cirillo C, Gigli S (2015). Enteric glia: a new player in inflammatory bowel diseases. Int J Immunopathol Pharmacol.

[134] Cirillo C, Sarnelli G, Esposito G (2009). Increased mucosal nitric oxide production in ulcerative colitis is mediated in part by the enteroglial-derived S100B protein. Neurogastroenterol Motil.

[135] Cirillo C, Sarnelli G, Esposito G (2011). S100B protein in the gut: the evidence for enteroglialsustained intestinal inflammation. World J Gastroenterol.

[136] Salhab WA, Perlman JM, Silver L, Sue Broyles R (2004). Necrotizing enterocolitis and neurodevelopmental outcome in extremely low birth weight infants. J Perinatol.

[137] Sonntag J, Grimmer I, Scholz T (2000). Growth and neurodevelopmental outcome of very low birthweight infants with necrotizing enterocolitis. Acta Paediatr.

[138] Wadhawan R, Oh W, Hintz SR (2014). Neurodevelopmental outcomes of extremely low birth weight infants with spontaneous intestinal perforation or surgical necrotizing enterocolitis. J Perinatol.

[139] Adesanya OA, O’Shea TM, Turner CS (2005). Intestinal perforation in very low birth weight infants: growth and neurodevelopment at 1 year of age. J Perinatol.

[140] Blakely ML, Tyson JE, Lally KP, et al (2006) Laparotomy versus peritoneal drainage for necrotizing enterocolitis or isolated intestinal perforation in extremely low birth weight infants: outcomes through 18 months adjusted age. Pediatrics 117:. 10.1542/peds.2005-1273 10.1542/peds.2005-1273 16549503

[141] Merhar SL, Ramos Y, Meinzen-Derr J, Kline-Fath BM (2014) Brain magnetic resonance imaging in infants with surgical necrotizing enterocolitis or spontaneous intestinal perforation versus medical necrotizing enterocolitis. J Pediatr 164:410–2.e1. 10.1016/j.jpeds.2013.09.055 10.1016/j.jpeds.2013.09.055 24210927

[142] Martin CR, Dammann O, Allred EN, et al (2010) Neurodevelopment of extremely preterm infants who had necrotizing enterocolitis with or without late bacteremia. J Pediatr 157:751–6.e1. 10.1016/j.jpeds.2010.05.042 10.1016/j.jpeds.2010.05.042 PMC2952050 20598317

[143] Hintz SR, Kendrick DE, Stoll BJ (2005). Neurodevelopmental and growth outcomes of extremely low birth weight infants after necrotizing enterocolitis. Pediatrics.

[144] Lenfestey MW, Neu J (2018). Gastrointestinal development: implications for management of preterm and term infants. Gastroenterol Clin N Am.

[145] Claud E (2009). Neonatal necrotizing enterocolitis – inflammation and intestinal immaturity. Antiinflamm Antiallergy Agents Med Chem.

[146] Heymans C, de Lange IH, Hütten MC (2020). Chronic intra-uterine ureaplasma parvum infection induces injury of the enteric nervous system in ovine fetuses. Front Immunol.

[147] Elgin TG, Fricke EM, Gong H et al (2019) Fetal exposure to maternal inflammation interrupts murine intestinal development and increases susceptibility to neonatal intestinal injury. DMM Dis Model Mech 12. 10.1242/dmm.040808 10.1242/dmm.040808 PMC6826024 31537532

[148] Lueschow SR, McElroy SJ (2020). The Paneth cell: the curator and defender of the immature small intestine. Front Immunol.

[149] Puri K, Taft DH, Ambalavanan N (2016). Association of chorioamnionitis with aberrant neonatal gut colonization and adverse clinical outcomes. PLoS One.

[150] Rizzetto L, Fava F, Tuohy KM, Selmi C (2018). Connecting the immune system, systemic chronic inflammation and the gut microbiome: the role of sex. J Autoimmun.

[151] Härtel C, Pagel J, Spiegler J (2017). Lactobacillus acidophilus/Bifidobacterium infantis probiotics are associated with increased growth of VLBWI among those exposed to antibiotics. Sci Rep.

[152] Schall KA, Thornton ME, Isani M (2017). Short bowel syndrome results in increased gene expression associated with proliferation, inflammation, bile acid synthesis and immune system activation: RNA sequencing a zebrafish SBS model. BMC Genomics.

[153] Plaza-Díaz J, Fontana L, Gil A (2018) Human milk oligosaccharides and immune system development. Nutrients 10 10.3390/nu10081038 PMC6116142 30096792

[154] Sonnenschein-van der Voort AMM, Arends LR, de Jongste JC (2014). Preterm birth, infant weight gain, and childhood asthma risk: a meta-analysis of 147,000 European children. J Allergy Clin Immunol.

[155] Hanski I, Von Hertzen L, Fyhrquist N (2012). Environmental biodiversity, human microbiota, and allergy are interrelated. Proc Natl Acad Sci U S A.

[156] Budden KF, Gellatly SL, Wood DLA (2017). Emerging pathogenic links between microbiota and the gut-lung axis. Nat Rev Microbiol.

[157] Lohmann P, Luna RA, Hollister EB (2014). The airway microbiome of intubated premature infants: characteristics and changes that predict the development of bronchopulmonary dysplasia. Pediatr Res.

[158] Wagner BD, Sontag MK, Harris JK, et al (2017) Airway microbial community turnover differs by BPD severity in ventilated preterm infants PLoS One 12:. 10.1371/journal.pone.0170120 10.1371/journal.pone.0170120 PMC5271346 28129336

[159] Wagner BD, Sontag MK, Harris JK (2017). Airway microbial community turnover differs by BPD severity in ventilated preterm infants. PLoS One.

[160] Lal CV, Travers C, Aghai ZH (2016). The airway microbiome at birth. Sci Rep.

[161] Davidson L, Berkelhamer S (2017). Bronchopulmonary dysplasia: chronic lung disease of infancy and long-term pulmonary outcomes. J Clin Med.

[162] Principi N, Di Pietro GM, Esposito S (2018). Bronchopulmonary dysplasia: clinical aspects and preventive and therapeutic strategies. J Transl Med.

[163] Speer CP (2006). Pulmonary inflammation and bronchopulmonary dysplasia. J Perinatol.

[164] Todd DA, Earl M, Lloyd J (1998). Cytological changes in endotracheal aspirates associated with chronic lung disease. Early Hum Dev.

[165] Kramer BW, Moss TJ, Willet KE (2001). Dose and time response after intraamniotic endotoxin in preterm lambs. Am J Respir Crit Care Med.

[166] Ikegami T, Tsuda A, Karube A (2000). Effects of intrauterine IL-6 and IL-8 on the expression of surfactant apoprotein mRNAs in the fetal rat lung. Eur J Obstet Gynecol Reprod Biol.

[167] Kallapur SG, Bachurski CJ, Le Cras TD (2004). Vascular changes after intra-amniotic endotoxin in preterm lamb lungs. Am J Physiol Cell Mol Physiol.

[168] Kallapur SG, Jobe AH, Ikegami M, Bachurski CJ (2003). Increased IP-10 and MIG expression after intra-amniotic endotoxin in preterm lamb lung. Am J Respir Crit Care Med.

[169] Prince LS, Okoh VO, Matalon S, Moninger TO (2004). Lipopolysaccharide increases alveolar type II cell number in fetal mouse lungs through toll-like receptor 4 and NF-kappaB. Am J Phys Lung Cell Mol Phys.

[170] Willet KE, Jobe AH, Ikegami M (2000). Antenatal endotoxin and glucocorticoid effects on lung morphometry in preterm lambs. Pediatr Res.

[171] Polglase GR, Hooper SB, Gill AW (2010). Intrauterine inflammation causes pulmonary hypertension and cardiovascular sequelae in preterm lambs. J Appl Physiol.

[172] Polglase GR, Nitsos I, Baburamani AA (2012). Inflammation in utero exacerbates ventilation-induced brain injury in preterm lambs. J Appl Physiol.

[173] Janér J, Lassus P, Haglund C (2006). Pulmonary vascular endothelial growth factor-C in development and lung injury in preterm infants. Am J Respir Crit Care Med.

[174] Kuo C, Lim S, King NJC (2011). Rhinovirus infection induces expression of airway remodelling factors in vitro and in vivo. Respirology.

[175] Meng F, Mambetsariev I, Tian Y (2015). Attenuation of lipopolysaccharide-induced lung vascular stiffening by lipoxin reduces lung inflammation. Am J Respir Cell Mol Biol.

[176] Marudamuthu AS, Bhandary YP, Shetty SK (2015). Role of the urokinase-fibrinolytic system in epithelial-mesenchymal transition during lung injury. Am J Pathol.

[177] Pan J, Zhan C, Yuan T (2018). Effects and molecular mechanisms of intrauterine infection/inflammation on lung development. Respir Res.

[178] Baker CD, Abman SH (2015). Impaired pulmonary vascular development in bronchopulmonary dysplasia. Neonatology.

[179] Mittendorf R, Covert R, Montag AG (2005). Special relationships between fetal inflammatory response syndrome and bronchopulmonary dysplasia in neonates. J Perinat Med.

[180] Gleditsch DD, Shornick LP, Van Steenwinckel J (2014). Maternal inflammation modulates infant immune response patterns to viral lung challenge in a murine model. Pediatr Res.

[181] Miao J, Zhang K, Lv M (2014). Circulating Th17 and Th1 cells expressing CD161 are associated with disease activity in rheumatoid arthritis. Scand J Rheumatol.

[182] Basdeo SA, Moran B, Cluxton D (2015). Polyfunctional, pathogenic CD161 ^+^ Th17 lineage cells are resistant to regulatory T cell–mediated suppression in the context of autoimmunity. J Immunol.

[183] Albertine KH, Jones GP, Starcher BC (1999). Chronic lung injury in preterm lambs. Disordered respiratory tract development. Am J Respir Crit Care Med.

[184] Coalson JJ, Winter VT, Siler-Khodr T, Yoder BA (1999). Neonatal chronic lung disease in extremely immature baboons. Am J Respir Crit Care Med.

[185] Tambunting F, Beharry KD, Hartleroad J (2005). Increased lung matrix metalloproteinase-9 levels in extremely premature baboons with bronchopulmonary dysplasia. Pediatr Pulmonol.

[186] Tambunting F, Beharry KDA, Waltzman J, Modanlou HD (2005). Impaired lung vascular endothelial growth factor in extremely premature baboons developing bronchopulmonary dysplasia/chronic lung disease. J Investig Med.

[187] Hillman NH, Polglase GR, Pillow JJ et al (2011) Inflammation and lung maturation from stretch injury in preterm fetal sheep. Am J Phys Lung Cell Mol Phys 300. 10.1152/ajplung.00294.2010 10.1152/ajplung.00294.2010 PMC3043810 21131401

[188] Wu S, Capasso L, Lessa A (2008). High tidal volume ventilation activates Smad2 and upregulates expression of connective tissue growth factor in newborn rat lung. Pediatr Res.

[189] Kroon AA, Wang J, Huang Z (2010). Inflammatory response to oxygen and endotoxin in newborn rat lung ventilated with low tidal volume. Pediatr Res.

[190] Thomson MA, Yoder BA, Winter VT (2004). Treatment of immature baboons for 28 days with early nasal continuous positive airway pressure. Am J Respir Crit Care Med.

[191] Thomson MA, Yoder BA, Winter VT (2006). Delayed extubation to nasal continuous positive airway pressure in the immature baboon model of bronchopulmonary dysplasia: lung clinical and pathological findings. Pediatrics.

[192] Leroy S, Caumette E, Waddington C, et al (2018) A time-based analysis of inflammation in infants at risk of bronchopulmonary dysplasia. J Pediatr 192:60-65.e1. 10.1016/j.jpeds.2017.09.011 10.1016/j.jpeds.2017.09.011 29092751

[193] Rudloff I, Cho SX, Bui CB (2017). Refining anti-inflammatory therapy strategies for bronchopulmonary dysplasia. J Cell Mol Med.

[194] Halliday HL, Ehrenkranz RA, Doyle LW (2010) Early (< 8 days) postnatal corticosteroids for preventing chronic lung disease in preterm infants. Cochrane database Syst rev CD001146. 10.1002/14651858.CD001146.pub3 10.1002/14651858.CD001146.pub3 20091516

[195] Reilly JM, Cunnion RE, Burch-Whitman C (1989). A circulating myocardial depressant substance is associated with cardiac dysfunction and peripheral hypoperfusion (lactic acidemia) in patients with septic shock. Chest.

[196] Suffredini AF, Fromm RE, Parker MM (1989). The cardiovascular response of normal humans to the administration of endotoxin. N Engl J Med.

[197] Sergi C, Shen F, Lim DW (2017). Cardiovascular dysfunction in sepsis at the dawn of emerging mediators. Biomed Pharmacother.

[198] Thornburg K, Jonker S, O’Tierney P (2011). Regulation of the cardiomyocyte population in the developing heart. Prog Biophys Mol Biol.

[199] Madsen-Bouterse SA, Romero R, Tarca AL (2010). The transcriptome of the fetal inflammatory response syndrome. Am J Reprod Immunol.

[200] Barker DJP, Osmond C, Winter PD (1989). Weight in infancy and death from ischaemic heart disease. Lancet.

[201] Parkinson JRC, Hyde MJ, Gale C (2013). Preterm birth and the metabolic syndrome in adult life: a systematic review and meta-analysis. Pediatrics.

[202] De Jong F, Monuteaux MC, Van Elburg RM (2012). Systematic review and meta-analysis of preterm birth and later systolic blood pressure. Hypertension.

[203] Barker DJ, Hales CN, Fall CH (1993). Type 2 (non-insulin-dependent) diabetes mellitus, hypertension and hyperlipidaemia (syndrome X): relation to reduced fetal growth. Diabetologia.

[204] Lewandowski AJ, Augustine D, Lamata P (2013). Preterm heart in adult life: cardiovascular magnetic resonance reveals distinct differences in left ventricular mass, geometry, and function. Circulation.

[205] Davis EF, Newton L, Lewandowski AJ (2012). Pre-eclampsia and offspring cardiovascular health: mechanistic insights from experimental studies. Clin Sci.

[206] Bonamy A-KE, Andolf E, Martin H, Norman M (2008). Preterm birth and carotid diameter and stiffness in childhood. Acta Paediatr.

[207] Belderbos ME, van Bleek GM, Levy O (2009). Skewed pattern of toll-like receptor 4-mediated cytokine production in human neonatal blood: low LPS-induced IL-12p70 and high IL-10 persist throughout the first month of life. Clin Immunol.

[208] Dembinski J, Behrendt D, Martini R (2003). Modulation of pro- and anti-inflammatory cytokine production in very preterm infants. Cytokine.

[209] Cedar H, Bergman Y (2011). Epigenetics of haematopoietic cell development. Nat Rev Immunol.

[210] Roseboom TJ, van der Meulen JH, Osmond C (2000). Coronary heart disease after prenatal exposure to the Dutch famine, 1944-45. Heart.

[211] Chistiakov DA, Orekhov AN, Bobryshev YV (2018). Chemokines and relevant microRNAs in the atherogenic process. Mini-Reviews Med Chem.

[212] Tare M, Bensley JG, Moss TJM (2014). Exposure to intrauterine inflammation leads to impaired function and altered structure in the preterm heart of fetal sheep. Clin Sci (Lond).

[213] Stock SJ, Patey O, Thilaganathan B (2017). Intrauterine Candida albicans infection causes systemic fetal candidiasis with progressive cardiac dysfunction in a sheep model of early pregnancy. Reprod Sci.

[214] Seehase M, Gantert M, Ladenburger A (2011). Myocardial response in preterm fetal sheep exposed to systemic endotoxinaemia. Pediatr Res.

[215] Hensler ME, Miyamoto S, Nizet V (2008). Group B streptococcal beta-hemolysin/cytolysin directly impairs cardiomyocyte viability and function. PLoS One.

[216] Barker DJ (1995). Fetal origins of coronary heart disease. BMJ.

[217] Barker DJ (1990). The fetal and infant origins of adult disease. BMJ.

[218] Baumgarten G, Knuefermann P, Schuhmacher G (2006). Toll-like receptor 4, nitric oxide, and myocardial depression in endotoxemia. Shock.

[219] Kramer BW, Ikegami M, Moss TJM (2005). Endotoxin-induced chorioamnionitis modulates innate immunity of monocytes in preterm sheep. Am J Respir Crit Care Med.

[220] Niu J, Azfer A, Kolattukudy PE (2008). Protection against lipopolysacharide-induced myocardial dysfunction in mice by cardiac-specific expression of soluble Fas. J Mol Cell Cardiol.

[221] Di Naro E, Cromi A, Ghezzi F, et al (2010) Myocardial dysfunction in fetuses exposed to intraamniotic infection: new insights from tissue Doppler and strain imaging. Am J Obstet Gynecol 203:459.e1–7. 10.1016/j.ajog.2010.06.033 10.1016/j.ajog.2010.06.033 20691411

[222] Romero R, Espinoza J, Goncalves L (2004). Fetal cardiac dysfunction in preterm premature rupture of membranes. J Matern Neonatal Med.

[223] Aye CYL, Lewandowski AJ, Lamata P (2017). Disproportionate cardiac hypertrophy during early postnatal development in infants born preterm. Pediatr Res.

[224] Velten M, Hutchinson KR, Gorr MW et al (2011) Systemic maternal inflammation and neonatal hyperoxia induces remodeling and left ventricular dysfunction in mice. PLoS One 6. 10.1371/journal.pone.0024544 10.1371/journal.pone.0024544 PMC3173376 21935422

[225] Burgner D, Liu R, Wake M, Uiterwaal CSP (2015). Do childhood infections contribute to adult cardiometabolic diseases?. Pediatr Infect Dis J.

[226] Hinchliffe SA, Sargent PH, Howard CV (1991). Human intrauterine renal growth expressed in absolute number of glomeruli assessed by the disector method and cavalieri principle. Lab Investig.

[227] Yzydorczyk C, Comte B, Cambonie G (2008). Neonatal oxygen exposure in rats leads to cardiovascular and renal alterations in adulthood. Hypertension.

[228] Rangel Frausto MS, Pittet D, Costigan M (1995). The natural history of the systemic inflammatory response syndrome (SIRS): a prospective study. JAMA J Am Med Assoc.

[229] Modena AB, Fieni S (2004). Amniotic fluid dynamics.

[230] Itabashi K, Ohno T, Nishida H (2003). Indomethacin responsiveness of patent ductus arteriosus and renal abnormalities in preterm infants treated with indomethacin. J Pediatr.

[231] Galinsky R, Moss TJM, Gubhaju L (2011). Effect of intra-amniotic lipopolysaccharide on nephron number in preterm fetal sheep. Am J Physiol Ren Physiol.

[232] Brenner BM, Garcia DL, Anderson S (1988). Glomeruli and blood pressure less of one, more the other?. Am J Hypertens.

[233] Klein SL, Flanagan KL (2016). Sex differences in immune responses. Nat Rev Immunol.

[234] Neubauer V, Griesmaier E, Ralser E, Kiechl-Kohlendorfer U (2012). The effect of sex on outcome of preterm infants - a population-based survey. Acta Paediatr Int J Paediatr.

[235] Klingström J, Lindgren T, Ahlm C (2008). Sex-dependent differences in plasma cytokine responses to hantavirus infection. Clin Vaccine Immunol.

[236] Libert C, Dejager L, Pinheiro I (2010). The X chromosome in immune functions: when a chromosome makes the difference. Nat Rev Immunol.

[237] Sawyer CC (2012). Child mortality estimation: estimating sex differences in childhood mortality since the 1970s. PLoS Med.

[238] Mendiratta DK, Rawat V, Thamke D (2006). Candida colonization in preterm babies admitted to neonatal intensive care unit in the rural setting. Indian J Med Microbiol.

[239] Nazir A, Masoodi T (2018). Spectrum of candidal species isolated from neonates admitted in an intensive care unit of teaching hospital of Kashmir, North India. J Lab Physicians.

[240] Kaur H, Chakrabarti A (2017). Strategies to reduce mortality in adult and neonatal candidemia in developing countries. J Fungi.

[241] Roy P, Kumar A, Kaur IR, Faridi MMA (2014). Gender differences in outcomes of low birth weight and preterm neonates: the male disadvantage. J Trop Pediatr.

[242] Minghetti L, Greco A, Zanardo V, Suppiej A (2013). Early-life sex-dependent vulnerability to oxidative stress: the natural twining model. J Matern Neonatal Med.

[243] Diaz-Castro J, Pulido-Moran M, Moreno-Fernandez J (2016). Gender specific differences in oxidative stress and inflammatory signaling in healthy term neonates and their mothers. Pediatr Res.

[244] Kim-Fine S, Regnault TRH, Lee JS (2012). Male gender promotes an increased inflammatory response to lipopolysaccharide in umbilical vein blood. J Matern Neonatal Med.

[245] Kosyreva AM (2014). The sex differences of morphology and immunology of SIRS of newborn Wistar rats. Int Sch Res Not.

[246] Cong X, Xu W, Janton S (2016). Gut microbiome developmental patterns in early life of preterm infants: impacts of feeding and gender. PLoS One.

[247] O’Driscoll DN, Greene CM, Molloy EJ (2017). Immune function?. A missing link in the gender disparity in preterm neonatal outcomes Expert Rev Clin Immunol.

[248] Kollmann TR, Kampmann B, Mazmanian SK (2017). Protecting the newborn and young infant from infectious diseases: lessons from immune ontogeny. Immunity.

[249] Harju M, Keski-Nisula L, Georgiadis L, et al (2014) The burden of childhood asthma and late preterm and early term births. J Pediatr 164:295–9.e1. 10.1016/j.jpeds.2013.09.057 10.1016/j.jpeds.2013.09.057 24210922

[250] Holsti A, Adamsson M, Hagglof B et al (2017) Chronic conditions and health care needs of adolescents born at 23 to 25 weeks’ gestation. Pediatrics 139. 10.1542/peds.2016-2215 10.1542/peds.2016-2215 28108580

[251] Fortmann I, Hartz A, Paul P (2018). Antifungal treatment and outcome in very low birth weight infants. Pediatr Infect Dis J.

[252] Kribs A, Roll C, Göpel W (2015). Nonintubated surfactant application vs conventional therapy in extremely preterm infants. JAMA Pediatr.

[253] Dorling J, Abbott J, Berrington J (2019). Controlled trial of two incremental milk-feeding rates in preterm infants. N Engl J Med.

[254] Renz H, Adkins BD, Bartfeld S (2018). The neonatal window of opportunity—early priming for life. J Allergy Clin Immunol.

[255] Lee AH, Shannon CP, Amenyogbe N (2019). Dynamic molecular changes during the first week of human life follow a robust developmental trajectory. Nat Commun.

[256] Olin A, Henckel E, Chen Y, et al (2018) Stereotypic immune system development in newborn children. Cell 174:1277-1292.e14. 10.1016/j.cell.2018.06.045 10.1016/j.cell.2018.06.045 PMC6108833 30142345

[257] Netea MG, Joosten LAB, Latz E, et al (2016) Trained immunity: a program of innate immune memory in health and disease. Science (80-. ). 352:427 10.1126/science.aaf1098 PMC5087274 27102489

[258] Ulas T, Pirr S, Fehlhaber B (2017). S100-alarmin-induced innate immune programming protects newborn infants from sepsis. Nat Immunol.

